# Event-Triggered Fault Estimation for Stochastic Systems over Multi-Hop Relay Networks with Randomly Occurring Sensor Nonlinearities and Packet Dropouts

**DOI:** 10.3390/s18030731

**Published:** 2018-02-28

**Authors:** Yunji Li, Li Peng

**Affiliations:** Key Laboratory of Advanced Process Control for Light Industry (Ministry of Education), Jiangnan University, Wuxi 214122, China; 7141905009@vip.jiangnan.edu.cn

**Keywords:** fault estimation, event-triggered data transmission, wireless sensors

## Abstract

Wireless sensors have many new applications where remote estimation is essential. Considering that a remote estimator is located far away from the process and the wireless transmission distance of sensor nodes is limited, sensor nodes always forward data packets to the remote estimator through a series of relays over a multi-hop link. In this paper, we consider a network with sensor nodes and relay nodes where the relay nodes can forward the estimated values to the remote estimator. An event-triggered remote estimator of state and fault with the corresponding data-forwarding scheme is investigated for stochastic systems subject to both randomly occurring nonlinearity and randomly occurring packet dropouts governed by Bernoulli-distributed sequences to achieve a trade-off between estimation accuracy and energy consumption. Recursive Riccati-like matrix equations are established to calculate the estimator gain to minimize an upper bound of the estimator error covariance. Subsequently, a sufficient condition and data-forwarding scheme are presented under which the error covariance is mean-square bounded in the multi-hop links with random packet dropouts. Furthermore, implementation issues of the theoretical results are discussed where a new data-forwarding communication protocol is designed. Finally, the effectiveness of the proposed algorithms and communication protocol are extensively evaluated using an experimental platform that was established for performance evaluation with a sensor and two relay nodes.

## 1. Introduction

The increased use of battery-powered wireless sensors can improve productivity and reduce installation costs in industrial processes. A variety of battery-powered wireless sensors span a wide range of applications including area detection, environmental sensing, industrial monitoring and control, etc. [1]. In these applications, data packet loss is often encountered in various practical environments owing to bandwidth constraints; and then, wireless sensors are practically often made under harsh environments including both uncontrollable elements and aggressive conditions [2]. In this case, estimator or observer results [3,4] based on the linear sensor may not provide a reliable solution and are not applicable. It should be pointed out that the size and costs of sensor nodes may result in constraints on resources such as energy, memory and computation speeds [5,6,7,8]. Such constraints may lead to development of new estimators and data transmission schemes against these constraints we mentioned above. On the other hand, it is also recognized that the failures of components appear always in many practical engineering systems. The occurrence of faults in sensors, actuator or process (plant) failures may drastically modify the system behavior, resulting in performance degradation or even instability. For the purpose of increasing the safety and reliability of networked controlled systems, fault diagnosis research and their applications to a wide range of industrial and commercial processes have been the subjects of intensive investigations over the past two decades [9,10,11,12]. Many fruitful results for a variety of systems have been reported [13,14,15,16,17,18,19,20,21].

In the past few years, a number of results related to state and/or fault estimation for a variety of systems with packet dropouts and/or sensor nonlinearities have been established in terms of all sorts of methods. Some examples are mentioned here. Linear and nonlinear estimation problems were tackled for missing measurements in [22], where the nonlinear function of sensor was modeled as a sector-bound condition. A robust filter was designed in [23] against the sensor saturation and the packet losses such that the filtering error dynamics was mean-square stable and the performance index was satisfied. The problem of asynchronous filtering was addressed in [24] for stochastic Markov jump systems with probabilistic occurring sensor nonlinearities. Recently, reducing the redundant data transmission operated by a wireless transmission module was referred to as an event-triggered data transmission scheme which was first presented in [25] on the concept of send-on-delta. This kind of transmission scheme taking system performance and energy conservation into account has been an active area of research and some outstanding results are made [26,27,28,29,30]. For instance, a modified Kalman filter using the send-on-delta method was designed in [26]. The study in [27] extended this to a varying-condition threshold in send-on-delta transmission scheme for stochastic nonlinear systems, where an easy-implemented recursive algorithm with consideration of linearization errors, time delays, and packet losses was derived. The work in [28] proposed optimal and suboptimal consensus filters with event-triggered communication protocols to achieve energy efficiency via reducing unnecessary interactions among the neighboring sensors. More related studies can be found in recent publications [31,32,33,34,35,36,37,38,39,40,41] and references therein.

As is mentioned above, most of the existing research is focused on single-hop networks where sensor nodes collect measurements and then wireless transmission modules in these sensor nodes transmit data directly to the remote estimator for estimating faults and states at each time. However, the sensor nodes cannot work properly once it exceeds its transmission distances. It can be also noted that the event-triggered sensor transmission scheme used in single-hop networks can simply be utilized in multi-hop networks case; that is, the sensors run transmission decision and relay nodes simply forward information to the remote estimator. Nevertheless, the relay nodes may not be able to complete the data-forwarding duty in the case of network failures (e.g., packet dropouts and jamming attacks). Furthermore, adding antennas may increase power consumption of the sensor nodes. Under these circumstances, there is no doubt that it is of significance to study remote estimation over the multi-hop relay networks.

In this paper, we consider the situation that a remote estimator is located far away from the process. A wireless sensor node has to forward its data packets to the remote estimator through a series of relay nodes over multi-hop links subject to random packet dropouts. This article will mainly focus on how to derive an event-triggered estimator of state and fault to against both randomly occurring nonlinearities and randomly occurring packet dropouts, and then how to design a data-forwarding scheme to realize a trade-off between estimation performance and energy consumption. In particular, we will design a new data-forwarding protocol that is verified on an experimental platform to ensure that sensors and a series of relay nodes can establish the multi-hop network perfectly when a “sleep” command is activated in the transmission module. The main contributions of this paper are summarized as follows:

(1) A co-design algorithm of event-triggered state and fault estimator is presented for a class of linear stochastic system, for the first time, to deal with the phenomena of simultaneous randomly occurring nonlinearity and randomly occurring packet dropouts, which reflects the reality closely. An upper bound of state and fault error covariances is minimized by appropriately designing the desired estimator gain.
(2) A Sufficient condition and a data-forwarding scheme are given such that the error covariance is mean-square bounded in the multi-hop relay links with random packet dropouts. Such data-forwarding scheme enables each relay node to forward the estimated values to the remote estimator.
(3) Implementation issues of the theoretical results are discussed. A new data-forwarding communication protocol that could be applied to our addressed topology is designed; this involves hardware design and the corresponding procedure implementation. The proposed communication protocol and theoretical results are verified in a classical industry-like process.

Nomenclature: Prob(x) means the occurrence probability of the event *x*. N and R denote the sets of natural and real numbers, respectively; $R^{m\times n}$ denotes the sets of *m* by *n* real-valued matrices, whereas $R^{n}$ is short for $R^{n\times 1}$; $R_{+}^{n\times n}$ and $R_{++}^{n\times n}$ are the sets of n×n positive semi-definite and positive definite matrices, respectively. When $X\in R_{+}^{n\times n}$, we simply write X≥0 (or X>0 if $X\in R_{++}^{n\times n})$. For $X\in R^{m\times n}$, $X^{T}$ denotes the transpose of *X*. For $x\in R^{m\times n}$, ${\left(x\right)}^{2}$ represents *x* by *x*. *I* is an identity matrix with appropriate dimensions. Furthermore, E(·), Var(·) and trace(·) denote the mathematical expectation, variance and the trace of a matrix, respectively.

## 2. Problem Statement

**Figure 1:**
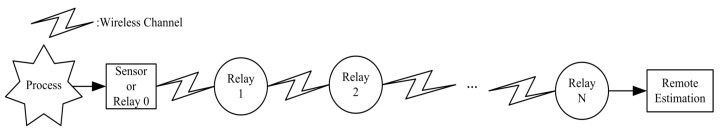
A block diagram of the system model.

A block diagram of a multi-hop relay network is given in Figure 1. The process is a discrete-time linear system defined on $k\in \left[0,L\right]$ that can be described by

(1) $${\bar{x}}_{k+1}=\bar{A}{\bar{x}}_{k}+{\bar{f}}_{k}+w_{k}$$

where a discrete time index k∈L and $L=\left\{0,1,\ldots \right\}$. The variables ${\bar{x}}_{k}\in R^{n}$ and ${\bar{f}}_{k}\in R^{n}$ are state vector and fault signal to be estimated, respectively. The noise signal $w_{k}\in R^{n}$ and is independent identically distributed (i.i.d) , satisfying Gaussian with zero-mean and known variance as follows

(2) $$E\left(w_{k}w_{k}^{T}\right)={\bar{Q}}_{w}\geq 0$$

The sensor measurement model with both randomly occurring nonlinearity and randomly occurring packet dropouts is described by

(3) $${\bar{y}}_{k}=\beta_{k}^{i}\left(\alpha_{k}\bar{C}{\bar{x}}_{k}+\left(1-\alpha_{k}\right)\bar{\phi }\left({\bar{x}}_{k}\right)\right)+v_{k}$$

where measurement output ${\bar{y}}_{k}\in R^{m}$ and measurement noise $v_{k}\in R^{m}$. It is another i.i.d noise signal satisfying Gaussian with zero-mean and known variance:

(4) $$E\left(v_{k}v_{k}^{T}\right)=R_{v}>0$$

where system matrix $\bar{A}$ and output matrix $\bar{C}$ are known with appropriate dimensions. Figure 1 illustrates that the data packets are transmitted to the remote estimator via wireless medium with successive *N* relay nodes. The current relay node will receive data packets only from its last node then forward data packets to next relay node. The sensor node is treated as relay 0 and other relay nodes are denoted as relay i(i=1,2,…,N). Additionally, let $\gamma_{k}^{i}$ be the decision variable: if $\gamma_{k}^{i}=1$, the data packets in the relay node *i* will be sent to the next relay node, and if $\gamma_{k}^{i}=0$, they will not be sent. The random variables $\alpha_{k}\in R$ and $\beta_{k}^{i}\in R$ are Bernoulli-distributed white sequences with the following probabilities.

(5) $$\left\{\begin{array}{c} \mathrm{Prob}\left\{\alpha_{k}=1\right\}=\alpha \\ \mathrm{Prob}\left\{\alpha_{k}=0\right\}=1-\alpha \end{array}\right.\;\mathrm{and}\;\left\{\begin{array}{c} \mathrm{Prob}\left\{\beta_{k}^{i}=1\right\}=\beta^{i}\\ \mathrm{Prob}\left\{\beta_{k}^{i}=0\right\}=1-\beta^{i} \end{array}\right.$$

where $\alpha ,\beta^{i}\in \left[0,1\right]$ are know constants. All random variables $\alpha_{k}$ and $\beta_{k}^{i}$ are assumed to be independent in *k* and uncorrelated with noise signals $w_{k}$ and $v_{k}$. The nonlinear function $\bar{\phi }\left(x_{k}\right)$ is further assumed to be known and analytic everywhere. The dynamic model of the fault vector ${\bar{f}}_{k}$ borrowed from [42,43] can be established as follows:

(6) $${\bar{f}}_{k+1}=\bar{M}{\bar{f}}_{k}$$

where $\bar{M}$ is a known matrix with appropriate dimensions.

**Remark** **1.** For the co-design problem of state and fault estimator using stochastic system model, a robust fault estimation filter design was proposed in terms of Riccati-like difference equations in [44,45]. Using the assumption that the sampling interval was sufficiently small, it was supposed that the fault difference item was too small to be neglected. However, in practice, faults always generate a great amplitude change of a certain time, especially when time-varying faults occur. Compared with the assumption in [44,45], it is clear that time-varying fault model described in Equation (6) covers the results of constant faults as a special case, which is less restrictive.

**Remark** **2.** The measurement model proposed in Equation (3) provides a unified framework to account for the phenomenon of both randomly occurring sensor nonlinearities and random packet dropouts. The stochastic variable $\alpha_{k}$ is indicated as the phenomenon of the probabilistic sensor nonlinearities, while the random variable $\beta_{k}^{i}$ is used to represent the nature of random packet dropouts. Specifically, if $\alpha_{k}=1$ and $\beta_{k}^{i}=1$, it means that the sensor work normally; if $\alpha_{k}=0$ and $\beta_{k}^{i}=1$, it can be seen that the sensor is subject to nonlinearity only; and if $\beta_{k}^{i}=0$, the measurement output contains the noise signal $v_{k}$ only, implying that the random packet dropouts occur.

By introducing a new vector $x_{k}=\left[\begin{array}{c} {\bar{x}}_{k}\\ {\bar{f}}_{k} \end{array}\right]$, we can rewrite Equations (1) and (3) as

(7) $$\begin{array}{cc} x_{k+1} & =Ax_{k}+Ww_{k}\\ y_{k} & =\beta_{k}^{i}\left(\alpha_{k}Cx_{k}+\left(1-\alpha_{k}\right)\phi \left(x_{k}\right)\right)+v_{k} \end{array}$$

where

(8) $$A=\left[\begin{array}{cc} \bar{A} & 0\\ 0 & \bar{M} \end{array}\right],\;W=\left[\begin{array}{c} I\\ 0 \end{array}\right],\;C=\left[\begin{array}{c} \bar{C}\\ 0 \end{array}\right],\;\phi \left(x_{k}\right)=\left[\begin{array}{c} \bar{\phi }\left({\bar{x}}_{k}\right)\\ 0 \end{array}\right]$$

Before giving the main results, the following lemma, which will be useful in this paper, needs to be introduced.

**Lemma** **1.** *(Lemma 1 [46]) Let A, D, E and F be real matrices of appropriate dimensions with $FF^{T}\leq I$. For any matrix $P=P^{T}>0$ and scalar ε>0 such that $\varepsilon^{-1}I-EPE^{T}>0$, then we have*

(9) $$\left(A+DFE\right)P{\left(A+DFE\right)}^{T}\leq A{\left(P^{-1}-\varepsilon E^{T}E\right)}^{-1}A^{T}+\varepsilon^{-1}DD^{T}$$

## 3. Main Results

### 3.1. A Co-Design Algorithm of Event-Triggered State and Fault Estimator

Based on the measurement $y_{k}$ mentioned before, the estimated variable ${\hat{x}}_{k}^{0}$ of the sensor node (or the relay node 0) can be recursively computed as follows.

(10) $${\hat{x}}_{k+1}^{0}=A{\hat{x}}_{k}^{0}+K_{k}\left(y_{k}-\alpha \beta^{0}C{\hat{x}}_{k}^{0}-\left(1-\alpha \right)\beta^{0}\phi \left({\hat{x}}_{k}^{0}\right)\right)$$

where $K_{k}$ is the estimator gain to be designed. Further, the estimation on the relay node *i* is suggested as follows:

(11) $${\hat{x}}_{k}^{i}=\gamma_{k}^{i-1}{\hat{x}}_{k}^{i-1}+\left(1-\gamma_{k}^{i-1}\right)A{\hat{x}}_{k-1}^{i}$$

while the corresponding estimation error covariance is given by

(12) $$P_{k}^{i}=\gamma_{k}^{i-1}P_{k}^{i-1}+\left(1-\gamma_{k}^{i-1}\right)\left(AP_{k-1}^{i}A^{T}+Q_{w}\right)$$

where estimation error $e_{k}^{i}=x_{k}-{\hat{x}}_{k}^{i}$, error covariance $P_{k}^{i}=E\left[e_{k}^{i}{\left(e_{k}^{i}\right)}^{T}\right]$ and $Q_{w}=W{\bar{Q}}_{w}W^{T}$.

**Remark** **3.** Traditionally, the remote estimator needs to know measurements collected by sensors at each time instant k. However, reduction of the number of relay-to-relay transmission actions has been adopted to make the relay nodes extend lifetime and save energy as much as possible. Under this circumstance, multi-hop links may create a problem: the measurements could not be obtained at each time instant and the estimated values could not be calculated by the remote estimator. Because the ultimate goal for remote estimation is to obtain estimated values at each time instant, it follows from Equation (11) that the relay nodes can forward “the estimated” values to the remote estimator.

The purpose of this section is to design an estimator of form Equation (10) for the stochastic system in Equation (1) and the sensor in Equation (3) with incomplete information (randomly occurring sensor nonlinearities and randomly occurring packet dropouts). More specifically, we are interested in looking for the filter parameter $K_{k}$ such that the following requirements are met simultaneously:

(a) For the phenomenon of packet loss and randomly occurring sensor nonlinearities, an upper bound of the error covariance $P_{k}^{0}$ is derived, i.e., there exists a sequence of positive-definite matrices ${\bar{P}}_{k}^{0}\left(0\leq k\leq L\right)$ that satisfies
  (13) $$E\left[\left(x_{k}-{\hat{x}}_{k}^{0}\right){\left(x_{k}-{\hat{x}}_{k}^{0}\right)}^{T}\right]\leq {\bar{P}}_{k}^{0}$$
(b) The sequence of upper bound ${\bar{P}}_{k}^{0}$ is minimized by the designed estimator gain $K_{k}$ through a recursive scheme.

Now we are in a position to obtain an upper bound of the error covariance $P_{k}^{0}$ in the following theorem.

**Theorem** **1.** *Consider the stochastic system described by Equation (1) with measurements in Equation (3) suffering from both packet loss and randomly occurring sensor nonlinearities. For a arbitrary positive constant γ and the given initial condition ${\bar{P}}_{0}^{0}=P_{0}^{0}>0$, if there exists $\gamma^{-1}I-L_{k}{\bar{P}}_{k}^{0}L_{k}^{T}>0$, then the error covariance $P_{k+1}^{0}\left(0\leq k\leq L\right)$ satisfies*

(14) $$P_{k+1}^{0}\leq {\bar{P}}_{k+1}^{0}$$

*where*

(15) $$\begin{array}{cc} {\bar{P}}_{k+1}^{0} & =\left(1+\varepsilon_{4}\right)\left(A-K_{k}\alpha \beta^{0}C\right){\bar{P}}_{k}^{0}{\left(A-K_{k}\alpha \beta^{0}C\right)}^{T}+Q_{w}\\ & +K_{k}\left(\left(\varepsilon_{1}+\varepsilon_{6}\right)C\left({\bar{P}}_{k}^{0}+{\hat{x}}_{k}^{0}{\left({\hat{x}}_{k}^{0}\right)}^{T}\right)C^{T}+R_{v}\right.+\left(\varepsilon_{3}+\varepsilon_{5}+\varepsilon_{6}\right)\varphi \left({\hat{x}}_{k}^{0}\right)+\\ & \left.\left(\varepsilon_{2}+\varepsilon_{4}+\varepsilon_{5}\right)\left(G_{k}{\left({\left({\bar{P}}_{k}^{0}\right)}^{-1}-\gamma L_{k}^{T}L_{k}\right)}^{-1}G_{k}^{T}+\gamma^{-1}H_{k}H_{k}^{T}\right)\right)K_{k}^{T} \end{array}$$

*with $\varepsilon_{1}=\alpha \beta^{0}-{\left(\alpha \beta^{0}\right)}^{2}$, $\varepsilon_{2}=\beta^{0}-\alpha \beta^{0}$, $\varepsilon_{3}=\left(2-\alpha \right)\beta^{0}+\left(2\alpha -2-\alpha^{2}\right){\left(\beta^{0}\right)}^{2}$, $\varepsilon_{4}=\left(1-\alpha \right)\beta^{0}$, $\varepsilon_{5}=\left(1-\alpha \right)\beta^{0}-{\left(1-\alpha \right)}^{2}{\left(\beta^{0}\right)}^{2}$, $\varepsilon_{6}=\left(\alpha -1\right)\alpha {\left(\beta^{0}\right)}^{2}$, $Q_{w}=W{\bar{Q}}_{w}W^{T}$ and $\varphi \left({\hat{x}}_{k}^{0}\right)=\phi \left({\hat{x}}_{k}^{0}\right)\phi^{T}\left({\hat{x}}_{k}^{0}\right)$.*

**Proof.**  First, the error dynamics of the addressed system are calculated by subtracting Equation (1) from Equation (10):

(16) $$\begin{array}{cc} e_{k+1}^{0} & =x_{k+1}-{\hat{x}}_{k}^{0}\\ & =Ax_{k}+Ww_{k}-A{\hat{x}}_{k}^{0}-K_{k}\left(y_{k}-\alpha \beta^{0}C{\hat{x}}_{k}^{0}-\left(1-\alpha \right)\beta^{0}\phi \left({\hat{x}}_{k}^{0}\right)\right)\\ & =Ae_{k}^{0}+Ww_{k}-K_{k}\left(\beta_{k}^{i}\left(\alpha_{k}Cx_{k}+\left(1-\alpha_{k}\right)\phi \left(x_{k}\right)\right)+v_{k}-\alpha \beta^{0}C{\hat{x}}_{k}^{0}-\left(1-\alpha \right)\beta^{0}\phi \left({\hat{x}}_{k}^{0}\right)\right)\\ & =Ae_{k}^{0}+Ww_{k}-K_{k}\left(\alpha \beta^{0}Ce_{k}^{0}+\left(\alpha_{k}\beta_{k}^{i}-\alpha \beta^{0}\right)Cx_{k}\right.\\ & \left.+\beta_{k}^{i}\left(1-\alpha_{k}\right)\phi \left(x_{k}\right)-\beta^{0}\left(1-\alpha \right)\phi \left({\hat{x}}_{k}^{0}\right)+v_{k}\right)\\ & =Ae_{k}^{0}+Ww_{k}-K_{k}\alpha \beta^{0}Ce_{k}^{0}-\left(\alpha_{k}\beta_{k}^{i}-\alpha \beta^{0}\right)K_{k}Cx_{k}-K_{k}v_{k}\\ & -K_{k}\left(\beta_{k}^{i}\left(1-\alpha_{k}\right)\phi \left(x_{k}\right)-\beta^{0}\left(1-\alpha \right)\phi \left({\hat{x}}_{k}^{0}\right)\right). \end{array}$$

With the help of results in [47] and Taylor series expansion to $\phi \left(x_{k}\right)$ around ${\hat{x}}_{k}^{0}$, we have

(17) $$\phi \left(x_{k}\right)=\phi \left({\hat{x}}_{k}^{0}\right)+G_{k}e_{k}^{0}+o\left(\left|e_{k}^{0}\right|\right),$$

where $G_{k}=\frac{\partial \phi \left(x_{k}\right)}{\partial x_{k}}\left|\begin{array}{c} x_{k}={\hat{x}}_{k}^{0} \end{array}\right.$ and $o\left(\left|e_{k}^{0}\right|\right)$ represented the first-order term of the Taylor series expansion. Moreover, the high-order term can be changed into the following form:

(18) $$o\left(\left|e_{k}^{0}\right|\right)=H_{k}N_{k}L_{k}e_{k}^{0}$$

where $H_{k}$ is a matrix with appropriate dimension that depends on the problem, $L_{k}$ is used to accommodate the estimator with a further extent of freedom, and $N_{k}$ is an unknown discrete-time matrix that stands for the error of linearization of model that requires $N_{k}N_{k}^{T}\leq I$. Inserting Equations (17) and (18) into Equation (16), the expression of estimation error can be expanded as

(19) $$\begin{array}{ll} e_{k+1}^{0} & =\left(A-K_{k}\alpha \beta^{0}C\right)e_{k}^{0}+Ww_{k}-\left(\alpha_{k}\beta_{k}^{i}-\alpha \beta^{0}\right)K_{k}Cx_{k}-K_{k}v_{k}\\ & -K_{k}\left(\beta_{k}^{i}\left(1-\alpha_{k}\right)\left(\phi \left({\hat{x}}_{k}^{0}\right)+G_{k}e_{k}^{0}+H_{k}N_{k}L_{k}e_{k}^{0}\right)-\beta^{0}\left(1-\alpha \right)\phi \left({\hat{x}}_{k}^{0}\right)\right)\\ & =\left(A-K_{k}\alpha \beta^{0}C\right)e_{k}^{0}+Ww_{k}-\left(\alpha_{k}\beta_{k}^{i}-\alpha \beta^{0}\right)K_{k}Cx_{k}-K_{k}v_{k}\\ & -\beta_{k}^{i}\left(1-\alpha_{k}\right)K_{k}\left(G_{k}+H_{k}N_{k}L_{k}\right)e_{k}^{0}-K_{k}\left(\beta_{k}^{i}\left(1-\alpha_{k}\right)\phi \left({\hat{x}}_{k}^{0}\right)-\beta^{0}\left(1-\alpha \right)\phi \left({\hat{x}}_{k}^{0}\right)\right)\\ & =\left(A-K_{k}\alpha \beta^{0}C\right)e_{k}^{0}+Ww_{k}-\left(\alpha_{k}\beta_{k}^{i}-\alpha \beta^{0}\right)K_{k}Cx_{k}-K_{k}v_{k}\\ & -\beta_{k}^{i}\left(1-\alpha_{k}\right)K_{k}\left(G_{k}+H_{k}N_{k}L_{k}\right)e_{k}^{0}-\left(\beta_{k}^{i}\left(1-\alpha_{k}\right)-\beta^{0}\left(1-\alpha \right)\right)K_{k}\phi \left({\hat{x}}_{k}^{0}\right). \end{array}$$

By the definition of error covariance $P_{k}^{0}$, it follows from Equation (16) that

(20) $$\begin{array}{ll} P_{k+1}^{0} & =E\left[e_{k+1}^{0}{\left(e_{k+1}^{0}\right)}^{T}\right]=\left(A-K_{k}\alpha \beta^{0}C\right)P_{k}^{0}{\left(A-K_{k}\alpha \beta^{0}C\right)}^{T}+Q_{w}+\varepsilon_{1}K_{k}CE\left[x_{k}x_{k}^{T}\right]C^{T}K_{k}^{T}\\ & +K_{k}R_{v}K_{k}^{T}+\varepsilon_{2}K_{k}\left(G_{k}+H_{k}N_{k}L_{k}\right)P_{k}^{0}{\left(G_{k}+H_{k}N_{k}L_{k}\right)}^{T}K_{k}^{T}+\varepsilon_{3}K_{k}\phi \left({\hat{x}}_{k}^{0}\right)\phi^{T}\left({\hat{x}}_{k}^{0}\right)K_{k}^{T}\\ & -\varepsilon_{4}\left(A-K_{k}\alpha \beta^{0}C\right)E\left[e_{k}^{0}{\left(e_{k}^{0}\right)}^{T}\right]{\left(G_{k}+H_{k}N_{k}L_{k}\right)}^{T}K_{k}^{T}\\ & -\varepsilon_{4}K_{k}\left(G_{k}+H_{k}N_{k}L_{k}\right)E\left[e_{k}^{0}{\left(e_{k}^{0}\right)}^{T}\right]{\left(A-K_{k}\alpha \beta^{0}C\right)}^{T}\\ & +\varepsilon_{5}K_{k}\left(G_{k}+H_{k}N_{k}L_{k}\right)E\left[e_{k}^{0}\phi^{T}\left({\hat{x}}_{k}^{0}\right)\right]K_{k}^{T}+\varepsilon_{5}K_{k}E\left[\phi \left({\hat{x}}_{k}^{0}\right){\left(e_{k}^{0}\right)}^{T}\right]{\left(G_{k}+H_{k}N_{k}L_{k}\right)}^{T}K_{k}^{T}\\ & +\varepsilon_{6}K_{k}CE\left[x_{k}\phi^{T}\left({\hat{x}}_{k}^{0}\right)\right]K_{k}^{T}+\varepsilon_{6}K_{k}E\left[\phi \left({\hat{x}}_{k}^{0}\right)x_{k}^{T}\right]C^{T}K_{k}^{T}. \end{array}$$

where $Q_{w}=W{\bar{Q}}_{w}W^{T}$, $\varepsilon_{1}=\alpha \beta^{0}-{\left(\alpha \beta^{0}\right)}^{2}$, $\varepsilon_{2}=\beta^{0}-\alpha \beta^{0}$, $\varepsilon_{3}=\left(2-\alpha \right)\beta^{0}+\left(2\alpha -2-\alpha^{2}\right){\left(\beta^{0}\right)}^{2}$, $\varepsilon_{4}=\left(1-\alpha \right)\beta^{0}$, $\varepsilon_{5}=\left(1-\alpha \right)\beta^{0}-{\left(1-\alpha \right)}^{2}{\left(\beta^{0}\right)}^{2}$ and $\varepsilon_{6}=\left(\alpha -1\right)\alpha {\left(\beta^{0}\right)}^{2}$. According to the initial condition ${\bar{P}}_{0}^{0}\geq P_{0}^{0}$, the upper bound of error covariance $P_{k+1}^{0}$ can be proved by induction. Let ${\bar{P}}_{k}^{0}\geq P_{k}^{0}$, we need to prove that ${\bar{P}}_{k+1}^{0}\geq P_{k+1}^{0}$. Using the elementary inequality $xy^{T}+yx^{T}\leq xx^{T}+yy^{T}$ and the results of Lemma 1, $P_{k+1}^{0}$ can be rewritten as

(21) $$\begin{array}{cc} P_{k+1}^{0} & \leq \left(1+\varepsilon_{4}\right)\left(A-K_{k}\alpha \beta^{0}C\right){\bar{P}}_{k}^{0}{\left(A-K_{k}\alpha \beta^{0}C\right)}^{T}+Q_{w}+\left(\varepsilon_{1}+\varepsilon_{6}\right)K_{k}CE\left[x_{k}x_{k}^{T}\right]C^{T}K_{k}^{T}\\ & +K_{k}R_{v}K_{k}^{T}+\left(\varepsilon_{2}+\varepsilon_{4}+\varepsilon_{5}\right)K_{k}\left(G_{k}+H_{k}N_{k}L_{k}\right){\bar{P}}_{k}^{0}{\left(G_{k}+H_{k}N_{k}L_{k}\right)}^{T}K_{k}^{T}\\ & +\left(\varepsilon_{3}+\varepsilon_{5}+\varepsilon_{6}\right)K_{k}\phi \left({\hat{x}}_{k}^{0}\right)\phi^{T}\left({\hat{x}}_{k}^{0}\right)K_{k}^{T}\\ & \leq \left(1+\varepsilon_{4}\right)\left(A-K_{k}\alpha \beta^{0}C\right){\bar{P}}_{k}^{0}{\left(A-K_{k}\alpha \beta^{0}C\right)}^{T}+Q_{w}+\left(\varepsilon_{1}+\varepsilon_{6}\right)K_{k}CE\left[x_{k}x_{k}^{T}\right]C^{T}K_{k}^{T}\\ & +\left(\varepsilon_{2}+\varepsilon_{4}+\varepsilon_{5}\right)K_{k}\left(G_{k}{\left({\left({\bar{P}}_{k}^{0}\right)}^{-1}-\gamma L_{k}^{T}L_{k}\right)}^{-1}G_{k}^{T}+\gamma^{-1}H_{k}H_{k}^{T}\right)K_{k}^{T}\\ & +K_{k}R_{v}K_{k}^{T}+\left(\varepsilon_{3}+\varepsilon_{5}+\varepsilon_{6}\right)K_{k}E\left[\phi \left({\hat{x}}_{k}^{0}\right)\phi^{T}\left({\hat{x}}_{k}^{0}\right)\right]K_{k}^{T}. \end{array}$$

Noticing the facts that $E\left[x_{k}x_{k}^{T}\right]=P_{k}^{0}+{\hat{x}}_{k}^{0}{\left({\hat{x}}_{k}^{0}\right)}^{T}$, the above Inequality (21) can be rewritten as follows:

(22) $$\begin{array}{cc} P_{k+1}^{0} & \leq \left(1+\varepsilon_{4}\right)\left(A-K_{k}\alpha \beta^{0}C\right){\bar{P}}_{k}^{0}{\left(A-K_{k}\alpha \beta^{0}C\right)}^{T}+Q_{w}+\left(\varepsilon_{1}+\varepsilon_{6}\right)K_{k}C\left({\bar{P}}_{k}^{0}+{\hat{x}}_{k}^{0}{\left({\hat{x}}_{k}^{0}\right)}^{T}\right)C^{T}K_{k}^{T}\\ & +\left(\varepsilon_{2}+\varepsilon_{4}+\varepsilon_{5}\right)K_{k}\left(G_{k}{\left({\left({\bar{P}}_{k}^{0}\right)}^{-1}-\gamma L_{k}^{T}L_{k}\right)}^{-1}G_{k}^{T}+\gamma^{-1}H_{k}H_{k}^{T}\right)K_{k}^{T}\\ & +K_{k}R_{v}K_{k}^{T}+\left(\varepsilon_{3}+\varepsilon_{5}+\varepsilon_{6}\right)K_{k}E\left[\phi \left({\hat{x}}_{k}^{0}\right)\phi^{T}\left({\hat{x}}_{k}^{0}\right)\right]K_{k}^{T}. \end{array}$$

Since the assumption that nonlinear function $\phi \left(x_{k}^{0}\right)$ is known and analytic everywhere, we can deduce that $E\left[\phi \left({\hat{x}}_{k}^{0}\right)\phi^{T}\left({\hat{x}}_{k}^{0}\right)\right]$ is calculable and further derive $P_{k+1}^{0}$ as the following form

(23) $$\begin{array}{cc} P_{k+1}^{0} & \leq \left(1+\varepsilon_{4}\right)\left(A-K_{k}\alpha \beta^{0}C\right){\bar{P}}_{k}^{0}{\left(A-K_{k}\alpha \beta^{0}C\right)}^{T}+Q_{w}+K_{k}\left(\left(\varepsilon_{1}+\varepsilon_{6}\right)C\left({\bar{P}}_{k}^{0}+{\hat{x}}_{k}^{0}{\left({\hat{x}}_{k}^{0}\right)}^{T}\right)C^{T}+\right.\\ & \left.\left(\varepsilon_{2}+\varepsilon_{4}+\varepsilon_{5}\right)\left(G_{k}{\left({\left({\bar{P}}_{k}^{0}\right)}^{-1}-\gamma L_{k}^{T}L_{k}\right)}^{-1}G_{k}^{T}+\gamma^{-1}H_{k}H_{k}^{T}\right)+\left(\varepsilon_{3}+\varepsilon_{5}+\varepsilon_{6}\right)\varphi \left({\hat{x}}_{k}^{0}\right)+R_{v}\right)K_{k}^{T}. \end{array}$$

which implies that Inequality (14) is true. ☐

In what follows, the gain matrix $K_{k}$ is determined by minimizing the upper bound of error covariance given by Equation (14).

**Theorem** **2.** *Consider the stochastic system described by Equation (1) with measurements in Equation (3) suffering from both packet dropouts and randomly occurring sensor nonlinearities. The gain matrix $K_{k}$ is given as follows*

(24) $$\begin{array}{cc} K_{k} & ={\tilde{\varepsilon }}_{1}A{\bar{P}}_{k}^{0}C^{T}\left[{\tilde{\varepsilon }}_{2}\right.C{\bar{P}}_{k}^{0}C^{T}+{\tilde{\varepsilon }}_{3}C{\hat{x}}_{k}^{0}{\left({\hat{x}}_{k}^{0}\right)}^{T}C^{T}+\\ & {\left.{\tilde{\varepsilon }}_{4}\left(G_{k}{\left({\left({\bar{P}}_{k}^{0}\right)}^{-1}-\gamma L_{k}^{T}L_{k}\right)}^{-1}G_{k}^{T}+\gamma^{-1}H_{k}H_{k}^{T}\right)+{\tilde{\varepsilon }}_{5}\varphi \left({\hat{x}}_{k}^{0}\right)+R_{v}\right]}^{-1} \end{array}$$

*where ${\tilde{\varepsilon }}_{1}=\left(1+\varepsilon_{4}\right)\alpha \beta^{0}$, ${\tilde{\varepsilon }}_{2}=\left(1+\varepsilon_{4}\right)\alpha^{2}{\left(\beta^{0}\right)}^{2}+{\tilde{\varepsilon }}_{3}$, ${\tilde{\varepsilon }}_{3}=\varepsilon_{1}+\varepsilon_{6}$, ${\tilde{\varepsilon }}_{4}=\varepsilon_{2}+\varepsilon_{4}+\varepsilon_{5}$ and ${\tilde{\varepsilon }}_{5}=\varepsilon_{3}+\varepsilon_{5}+\varepsilon_{6}$. Furthermore, the upper bound of the estimator error covariance ${\bar{P}}_{k+1}^{0}$ is recursively calculated by Riccati-like difference Equation (15).*

**Proof.**  We are ready to show that the gain described by Equation (24) is optimal in the sense that minimizes the upper bound ${\bar{P}}_{k+1}^{0}$. Note that three terms in Equation (15) are quadratic in $K_{k}$. The matrix differentiation formulas may be applied to Equation (15). Now differentiate $trace\left({\bar{P}}_{k+1}^{0}\right)$ with respect to $K_{k}$. The result is

(25) $$\begin{array}{cc} \frac{\partial \left(trace\left({\bar{P}}_{k+1}^{0}\right)\right)}{\partial \left(K_{k}\right)} & =\left(1+\varepsilon_{4}\right)\left(A-K_{k}\alpha \beta^{0}C\right){\bar{P}}_{k}^{0}{\left(A-K_{k}\alpha \beta^{0}C\right)}^{T}+Q_{w}+\\ & K_{k}\left(\left(\varepsilon_{1}+\varepsilon_{6}\right)C\left({\bar{P}}_{k}^{0}+{\hat{x}}_{k}^{0}{\left({\hat{x}}_{k}^{0}\right)}^{T}\right)C^{T}+\right.\left(\varepsilon_{3}+\varepsilon_{5}+\varepsilon_{6}\right)\varphi \left({\hat{x}}_{k}^{0}\right)+R_{v}+\\ & \left.\left(\varepsilon_{2}+\varepsilon_{4}+\varepsilon_{5}\right)\left(G_{k}{\left({\left({\bar{P}}_{k}^{0}\right)}^{-1}-\gamma L_{k}^{T}L_{k}\right)}^{-1}G_{k}^{T}+\gamma^{-1}H_{k}H_{k}^{T}\right)\right)K_{k}^{T}. \end{array}$$

We set the derivative equal to zero and the optimal gain is solved as follows

(26) $$\begin{array}{cc} K_{k} & ={\tilde{\varepsilon }}_{1}A{\bar{P}}_{k}^{0}C^{T}\left[{\tilde{\varepsilon }}_{2}\right.C{\bar{P}}_{k}^{0}C^{T}+{\tilde{\varepsilon }}_{3}C{\hat{x}}_{k}^{0}{\left({\hat{x}}_{k}^{0}\right)}^{T}C^{T}+\\ & {\left.{\tilde{\varepsilon }}_{4}\left(G_{k}{\left({\left({\bar{P}}_{k}^{0}\right)}^{-1}-\gamma L_{k}^{T}L_{k}\right)}^{-1}G_{k}^{T}+\gamma^{-1}H_{k}H_{k}^{T}\right)+{\tilde{\varepsilon }}_{5}\varphi \left({\hat{x}}_{k}^{0}\right)+R_{v}\right]}^{-1} \end{array}$$

with ${\tilde{\varepsilon }}_{1}=\left(1+\varepsilon_{4}\right)\alpha \beta^{0}$, ${\tilde{\varepsilon }}_{2}=\left(1+\varepsilon_{4}\right)\alpha^{2}{\left(\beta^{0}\right)}^{2}+{\tilde{\varepsilon }}_{3}$, ${\tilde{\varepsilon }}_{3}=\varepsilon_{1}+\varepsilon_{6}$, ${\tilde{\varepsilon }}_{4}=\varepsilon_{2}+\varepsilon_{4}+\varepsilon_{5}$ and ${\tilde{\varepsilon }}_{5}=\varepsilon_{3}+\varepsilon_{5}+\varepsilon_{6}$. which is as same as (24). It is clear that the estimator gain is optimal that minimizes the upper bound ${\bar{P}}_{k+1}^{0}$ for the estimator error covariance. ☐

### 3.2. Data Forwarding with Packet Dropouts

Thus far, we have derived an upper bound of the estimator error covariance and such an upper bound is subsequently minimized by properly designing the estimator gain. However, as shown in Section 3.1, we only consider the case of data packet dropouts in the sensor transmission stage. The problem on data packet loss is neglected in the multi-hop links. In the following, the mean-square boundedness of the error covariance $P_{k}^{i}$ will be presented.

**Theorem** **3.** *Consider the relay node i and the stochastic system described by Equation (1) subject to random packet loss in the multi-hop links. Let $\rho_{s}\left(A\right)$ be s-th eigenvalue of matrix A and s=1,⋯,n. If system matrix A is unstable and satisfies $\left|\rho_{s}\left(A\right)\right|<\frac{1}{\sqrt{\beta^{i}}}$, then the error covariance $P_{k}^{i}$ is mean-square bounded, namely, $E\left(P_{k}^{i}\right)\leq \Theta$, where* Θ *is a unique positive solution.*

**Proof.**  The upper bound of the error covariance $P_{k}^{i}$ in the relay node *i* is updated according to Equations (12) and (14), and then, by taking expectation on both sides, we obtain

(27) $$E\left({\bar{P}}_{k}^{i}\right)=\beta^{i}\left(AE\left({\bar{P}}_{k-1}^{i}\right)A^{T}+Q_{w}\right)+\left(1-\beta^{i}\right)E\left({\bar{P}}_{k}^{i-1}\right).$$

The differences of expectations between two adjacent sampling instants can be derived as follows

(28) $$\begin{array}{cc} E\left({\bar{P}}_{1}^{i}\right)-E\left({\bar{P}}_{0}^{i}\right) & =\beta^{i}\left(AE\left({\bar{P}}_{0}^{i}\right)A^{T}+Q_{w}\right)+\left(1-\beta^{i}\right)E\left({\bar{P}}_{1}^{i-1}\right)-E\left({\bar{P}}_{0}^{i}\right)\\ & =\left(1-\beta^{i}\right)\left(E\left({\bar{P}}_{1}^{i-1}\right)-E\left({\bar{P}}_{0}^{i-1}\right)\right)+\beta^{i}\left(AE\left({\bar{P}}_{0}^{i}\right)A^{T}+Q_{w}-E\left({\bar{P}}_{0}^{i}\right)\right)\\ & =\left(1-\beta^{i}\right)\left(E\left({\bar{P}}_{1}^{i-1}\right)-P_{0}^{0}\right)+\beta^{i}\left(AP_{0}^{0}A^{T}+Q_{w}-P_{0}^{0}\right) \end{array}$$

where the initial condition ${\bar{P}}_{0}^{i}=P_{0}^{0}>0$. According to the considered topology given in Figure 1 and the unstable system matrix *A*, it is shown that

(29) $$\begin{array}{c} \mathrm{if}\;E\left({\bar{P}}_{1}^{i-1}\right)=P_{0}^{0},\;\mathrm{we}\mathrm{have}\;E\left({\bar{P}}_{1}^{i}\right)-E\left({\bar{P}}_{0}^{i}\right)=\beta^{i}\left(AP_{0}^{0}A^{T}+Q_{w}-P_{0}^{0}\right);\\ \mathrm{else}\mathrm{if}\;E\left({\bar{P}}_{1}^{i-1}\right)=AP_{0}^{0}A^{T}+Q_{w},\;\mathrm{we}\mathrm{have}\;E\left({\bar{P}}_{1}^{i}\right)-E\left({\bar{P}}_{0}^{i}\right)=AP_{0}^{0}A^{T}+Q_{w}-P_{0}^{0}. \end{array}$$

Then, from the above equalities in Equation (29), we can infer that $E\left({\bar{P}}_{1}^{i}\right)>E\left({\bar{P}}_{0}^{i}\right)$ via the Lemma 2.2 presented in [48], which implies that $E\left({\bar{P}}_{1}^{i}\right)\leq \Theta_{1}$, where $\Theta_{1}>0$. Utilizing the induction method and the continuity of Equation (27), we can know that $E\left({\bar{P}}_{k}^{i}\right)\leq \Theta$, where Θ>0. Further, let us denote $P_{\infty }^{i}$ as the steady-state value for $E\left({\bar{P}}_{k}^{i}\right)$ in the current relay node *i*, and $P_{\infty }^{i}$ is the solution of the following matrix equation.

(30) $$\beta^{i}AP_{\infty }^{i}A^{T}-P_{\infty }^{i}=-\beta^{i}Q_{w}-\left(1-\beta^{i}\right)E\left({\bar{P}}_{k}^{i-1}\right)$$

where $E\left({\bar{P}}_{k}^{i-1}\right)>0$. This is equivalent to an extended Lyapunov equation and has a unique positive solution if

(31) $$\left|\rho_{s}\left(A\right)\right|<\frac{1}{\sqrt{\beta^{i}}}.$$

As a result, $E\left(P_{k}^{i}\right)\leq \Theta$, where Θ is a unique positive solution. It can be concluded that the boundedness and convergence of $E\left(P_{k}^{i}\right)$ is guaranteed. ☐

**Remark** **4.** In fact, from Equations (29) and (28), it is obviously known that $E\left({\bar{P}}_{1}^{i}\right)\leq E\left({\bar{P}}_{0}^{i}\right)=P_{0}^{0}$, when system matrix A is stable. Therefore, the error covariance $P_{k}^{i}$ is also mean-square bounded if the stochastic system presented by Equation (1) is stable.

**Remark** **5.** Due to the random packet dropouts, the error covariance $P_{k}^{i}$ is time-varying for any given positive initial state. However, $P_{k}^{i}$ is bounded with probability if $E\left(P_{k}^{i}\right)$ is bounded [49]. Therefore, $E\left(P_{k}^{i}\right)\leq \Theta$ with Θ>0 can be considered that the estimation error is mean square stable.

Although many event-triggered sensor schedules (e.g., [50,51]) can be utilized in the multi-hop networks, wireless communication network failures make the relay nodes unable to complete the data-forwarding tasks. Thus, it is necessary to design an energy-efficient data-forwarding scheme for relay nodes against the situation of network data dropouts.

**Theorem** **4.** *Given that a positive constant $\delta^{i}<\infty$, if the following event condition of the relay node i*

(32) $$trace\left(\left|{\bar{P}}_{k}^{i}-{\bar{P}}_{k-1}^{i}\right|\right)\leq \delta^{i}$$

*is satisfied, where $\left|\cdot \right|$ stands for the absolute value, then the proposed estimator in Equation (11) can ensure that $trace\left(E\left({\bar{P}}_{k}^{i}\right)\right)$ is bounded by $trace\left(E\left({\bar{P}}_{k}^{i}\right)\right)\leq \Omega$, where Ω is a unique positive solution.*

**Proof.**  Let us start to recall the following expression for $E\left({\bar{P}}_{k}^{i}\right)$ in Theorem 3

(33) $$E\left({\bar{P}}_{k}^{i}\right)=\beta^{i}\left(AE\left({\bar{P}}_{k-1}^{i}\right)A^{T}+Q_{w}\right)+\left(1-\beta^{i}\right)E\left({\bar{P}}_{k}^{i-1}\right).$$

Using the property of matrix trace, the above Equation (33) becomes

(34) $$trace\left(E\left({\bar{P}}_{k}^{i}\right)\right)=\beta^{i}trace\left(\left(AE\left({\bar{P}}_{k-1}^{i}\right)A^{T}+Q_{w}\right)\right)+\left(1-\beta^{i}\right)trace\left(E\left({\bar{P}}_{k}^{i-1}\right)\right).$$

For the sequence $trace\left(E\left({\bar{P}}_{k}^{i}\right)\right)$, we have

(35) $$\begin{array}{c} E\left(trace\left({\bar{P}}_{1}^{i}\right)\right)-E\left(trace\left({\bar{P}}_{0}^{i}\right)\right)\\ =\beta^{i}trace\left(AE\left({\bar{P}}_{0}^{i}\right)A^{T}+Q_{w}\right)+\left(1-\beta^{i}\right)trace\left(E\left({\bar{P}}_{1}^{i-1}\right)-E\left({\bar{P}}_{0}^{i}\right)\right)\\ =\left(1-\beta^{i}\right)\left(traceE\left({\bar{P}}_{1}^{i-1}-{\bar{P}}_{0}^{i-1}\right)\right)+\beta^{i}trace\left(AE\left({\bar{P}}_{0}^{i}\right)A^{T}+Q_{w}-E\left({\bar{P}}_{0}^{i}\right)\right)\\ \leq \left(1-\beta^{i}\right)trace\left(\left|{\bar{P}}_{k}^{i}-{\bar{P}}_{k-1}^{i}\right|\right)+\beta^{i}trace\left(AE\left({\bar{P}}_{0}^{i}\right)A^{T}+Q_{w}-E\left({\bar{P}}_{0}^{i}\right)\right) \end{array}$$

where $\left|\cdot \right|$ represents the absolute value. Then, substituting the event condition in Equation (32) into Equation (35) yields

(36) $$E\left(trace\left({\bar{P}}_{1}^{i}\right)\right)-E\left(trace\left({\bar{P}}_{0}^{i}\right)\right)\leq \beta^{i}trace\left(AE\left({\bar{P}}_{0}^{i}\right)A^{T}+Q_{w}-E\left({\bar{P}}_{0}^{i}\right)\right).$$

The following proof of Theorem 4 is similar to that of Theorem 3. The detailed proof is thus omitted. ☐

**Algorithm 1** Event-triggered data-forwarding schemeAt each time instant *k*, the relay node *i* executes: initialization ${\hat{x}}_{0}^{i}$ and ${\bar{P}}_{0}^{i}=P_{0}^{0}$;
1:**while**$\beta_{k}^{i}=0$ **or** $\gamma_{k}^{i-1}=0$, the relay node *i* cannot receive the estimated value due to data packet dropouts or event condition (32) **do**2:    ${\hat{x}}_{k}^{i}=A{\hat{x}}_{k-1}^{i}$;3:    ${\bar{P}}_{k}^{i}=A{\bar{P}}_{k-1}^{i}A^{T}+Q_{w}$;4:    **if** $trace\left(\left|{\bar{P}}_{k}^{i}-{\bar{P}}_{k-1}^{i}\right|\right)>\delta^{i}$ **then**5:        $\gamma_{k}^{i}=1$, the current relay node *i* can send the data packet;6:    **else**7:        $\gamma_{k}^{i}=0$, the current relay node *i* is not allowed to send the data packet;8:    **end if**9:**end while**10:${\hat{x}}_{k}^{i}={\hat{x}}_{k}^{i-1}$;11:${\bar{P}}_{k}^{i}={\bar{P}}_{k}^{i-1}$;12:$\beta_{k}^{i}=1$ **and** $\gamma_{k}^{i-1}=1$, declaring that the relay node *i* has successfully received data packets. For the purpose of achieving more accurate estimation for remote estimator, data packets of the relay node *i* are sent to the next relay node without entering the event-triggered decision.

We now elaborate this scheme described in Algorithm 1 for relay node *i*. First of all, the measurements are collected locally at each time instant, then the state values are estimated by a steady-state Kalman filter. Next, the relay node *i* will forward the estimated state values to the next relay node. If $\gamma_{k}^{i-1}=1$ and $\beta_{k}^{i}=1$, the relay node *i* will successfully receive the estimated state values from relay node i−1 at time instant *k*, i.e., ${\hat{x}}_{k}^{i}={\hat{x}}_{k}^{i-1}$ and the corresponding error variance can be calculated as ${\bar{P}}_{k}^{i}={\bar{P}}_{k}^{i-1}$. To achieve more accurate estimates of system state, the relay node *i* will forward data packets to the next relay node without entering the event-triggered decision rule. Whereas, if $\gamma_{k}^{i-1}=0$ or $\beta_{k}^{i}=0$ at time instant *k*, relay node i−1 will not send the estimated state values to relay node *i* for energy conservation (or relay node *i* cannot receive the data packets due to data losses). Without state information from relay node i−1, the estimated state values and error variance at relay node *i* will be updated as follows: ${\hat{x}}_{k}^{i}=A{\hat{x}}_{k-1}^{i}$, and ${\bar{P}}_{k}^{i}=A{\bar{P}}_{k-1}^{i}A^{T}+Q_{w}$. Then, the event-triggered decision rule will determine whether the relay node *i* will send the current estimated state value ${\hat{x}}_{k}^{i}$ to the next relay node.

## 4. Experimental Verification

In this section, the effectiveness of the proposed theoretical algorithm will be evaluated on a test bed that is a scale-down industrial process of twin water tanks. Based on the architecture of Figure 1, a sensor node (Node 1) and two relay nodes (Node 2 and Node 3) are designed to construct a multi-hop network transmitting water level information from Node 1 to Node 2 and to Node 3. At Node 3, the information on water level will be fed to a remote computer. This section is organized as follows. A new transmission protocol including the implementation of hardware and the corresponding procedure is presented in Section 4.1. Section 4.2 introduces system description and modeling. Experimental results on estimation quality and energy conservation are obtained in Section 4.3.

To verify the effectiveness of our proposed data-forwarding scheme, we shall present a new transmission protocol in the following section.

### 4.1. A New Transmission Protocol for Data Forwarding Scheme

In the most industrial applications, a wireless transmission module (WTM) always consumes more energy than a computation module. This is why we have designed data-forwarding scheme to reduce the amount of time for sending and receiving data, making the lifetime of the wireless node longer. However, stopping communication does not mean stopping energy consumption. It is because the WTM of each node keeps monitoring whether the data has arrived or not. Although the characteristic of the WTM can make it sleep to achieve the result of energy conservation in the single-hop wireless networks [52], the wireless transmission technology may not allow us to obtain such a sleeping capability in the multi-hop networks. For example, two nodes including station (STA) mode and access point (AP) mode have been embedded in the Wi-fi technology and they have to exist in the relay node. However, the WTM chosen as AP mode will spend a lot of time waking up (or even cannot wake up) once it enters a sleep state. It will extend the transmission time and lead to limited applications in real-world applications. The ZigBee communication technology cannot be applied to the network topology described in Figure 1 because the coordinator and router, which have to be added as a relay node in the multi-hop network, cannot go to sleep. In addition, the Bluetooth technique is not qualified as the WTM of relay nodes due to its long matching time and limited transmission distance. All of these motivate us to come up with a new transmission protocol suitable for any data-forwarding schemes in multi-hop relay networks.

First, we introduce the components of each relay node: (i) the wireless transmission module forwards the data packets between the relay nodes; (ii) the computation module determines when to forward data packets via our forwarding scheme; (iii) the switching module turns off and on the power of WTM; and (iv) the transmitter and receiver are a pair of wireless transceivers to distinguish them from the WTM. The transmitter and receiver are used as a medium to wake up WTM quickly and to ensure the network connectivity when the WTM is commanded into a sleep mode.

The procedure of this new transmission protocol is now presented in Algorithm 2.

**Algorithm 2** The implementation steps for the new transmission protocolWhen the data packet is requested to be sent from the relay node *i* to the relay node i+1, the following steps are performed: **Step 1**: For relay node *i*, the computation module sends a specified digital signal to the transmitter through I/O ports. **Step 2**: For relay node *i*, the switching module turns on the power of WTM. **Step 3**: The transmitter of relay node *i* sends a signal to the receiver of relay node i+1. **Step 4**: For relay node i+1, the receiver sends a specified digital signal to wake up the computation module by I/O ports. **Step 5**: For relay node i+1, the computation module requires switching module to power on the WTM. **Step 6**: The WTM of relay node *i* forwards data packets to the WTM of relay node i+1. **Step 7**: For relay node *i*, the switching module turns off the power of WTM after the end of transmission. **end**

#### 4.1.1. Hardware Design for the Experiment

**Figure 2:**
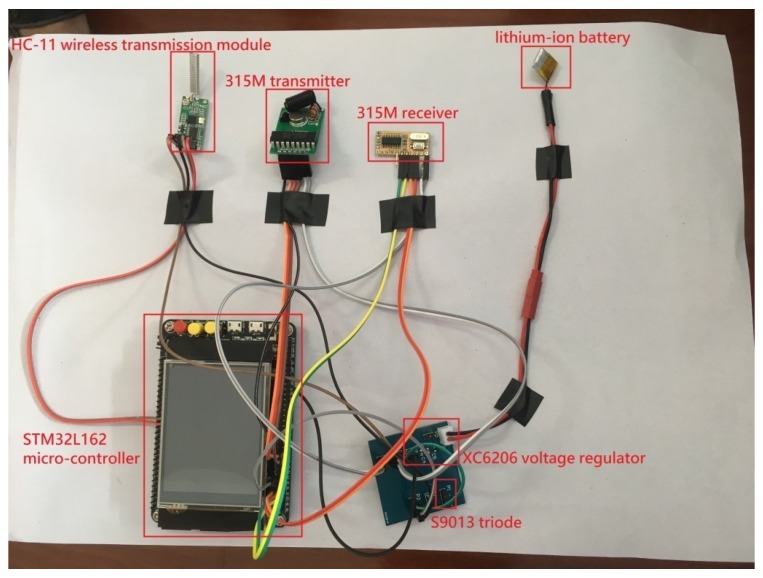
The physical connection diagram of Node 2.

Node 1 acts as the head node, Nodes 2 and 3 together construct a multi-hop network for verification of our theoretical results in this experiment. The physical connection diagram in Figure 2 is a photograph of the components in Node 2. Due to the limited space in this paper, the structures of Node 1 and Node 3 are omitted. They are similar to the structure of Node 1 except that the receiver and transmitter ignored in the Node 1 and Node 3, respectively. As shown in Figure 2, the node contains the following components: an STM32L162ZD micro-controller [53] (STM32, Geneva, Switzerland) including an ARM cortexTM-M3 CPU, a 384 Kbytes Flash memory, and a 48 Kbytes RAM that allows us to use it as a computation module; and an HC-11 [54] (also called 433 Mhz UART serial wireless transceiver module) with simple and flexible operation is selected as the WTM. Furthermore, the corresponding switching module is an S9013 NPN type triode and the power management system is composed of an X6206 voltage regulator and a Lithium-ion battery. In particular, the reason why we choose 315M transmitter and receiver is due to their extreme low power consumption. Even though its transmission rate is very limited, the electric current of idle state is approximately 0 mA, and the electric current in transmission state is lower than 2 mA.

#### 4.1.2. Implementation of the Experiment

To implement this experiment, two scenarios will be discussed in Algorithms 3 and 4. If the transmission commands are calculated by the STM32 using our designed forwarding scheme, the active mode is executed and the sleep mode is activated otherwise.

**Algorithm 3** The active mode for Node *i*When Node *i* sends the data packet to Node i+1, the following steps will be performed: **Step 1**: For Node *i*: STM32 sends a signal to 315M transmitter and activates HC-11. **Step 2**: For Node i+1: 315M receiver activates STM32 then activates HC-11. **Step 3**: Node *i* forwards data packets to Node i+1. **Step 4**: For Node *i*: turns off HC-11. **end**

**Algorithm 4** The sleep mode for Node *i*When Node *i* is not allowed to send the data packet to Node i+1, the following steps will be performed: **Step 1**: For Node *i*: STM32 and 315M transmitters enter an idle state and the HC-11 is not turned on. **Step 2**: For Node i+1: STM32 calculates the corresponding decision to determine whether or not sending data packets based on the proposed data-forwarding scheme. The 315M receiver enters an idle state then HC-11 is not turned on. **end**

Additionally, the received data packets may contain incomplete data packets (or data packets with error information) due to network failures, so a data validation algorithm is presented in Algorithm 5 which also reduce the probability of data-packet loss to some extent. We now introduce two indicators $flag1\in \left\{success,\;failure\right\}$ and $flag2\in \left\{success,\;failure\right\}$. Either flag1=failure or flag2=failure, the re-transmission commands sent by Node 2 (or Node 3) will be fed back to Node 1 (or Node 2). Conversely, both flag1=success and flag2=success, the end command will be executed.

**Algorithm 5** Data validationWhen sending the data packets from Node 1 to Node 2 (or from Node 2 to Node 3), the following procedures will be executed: initialization flag1=flag2=failure;
1:**while** flag1=failure **or** flag2=failure **do**2:    **if** Node 2 (or Node 3) does not receive the data packets, **then**3:        flag1=failure, Node 2 (or Node 3) sends the retransmission command to Node 1 (or Node 2);4:    **else if** data-packet length is incomplete **then**5:        flag1=success;6:        flag2=failure, Node 2 (or Node 3) sends the re-transmission command to Node 1 (or Node 2);7:    **else**8:        flag1=success;9:        flag2=success, Node 2 (or Node 3) sends the end command to Node 1 (or Node 2);10:    **end if**11:**end while**


### 4.2. System Description and Modeling of the Twin Water-Tank System

**Figure 3:**
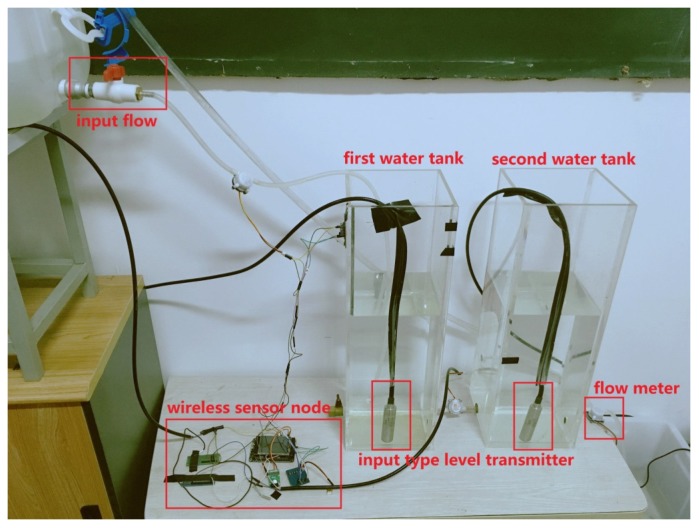
The components of twin water-tanks.

In this subsection, the feasibility and practicality of the proposed theoretical results and the transmission protocol will be examined on a continuous-time linear model [55]. Figure 3 is a photograph of the architecture of the twin water-tank system including two small tanks and a reservoir. The system state-space equations are described as follows.

(37) $$\left[\begin{array}{c} {\dot{h}}^{\left(1\right)}\\ {\dot{h}}^{\left(2\right)} \end{array}\right]=\left[\begin{array}{cc} -\frac{1}{A^{\left(1\right)}r^{\left(1\right)}} & \frac{1}{A^{\left(1\right)}r^{\left(1\right)}}\\ \frac{1}{A^{\left(2\right)}r^{\left(1\right)}} & -\left(\frac{1}{A^{\left(2\right)}r^{\left(1\right)}}+\frac{1}{A^{\left(2\right)}r^{\left(2\right)}}\right) \end{array}\right]\left[\begin{array}{c} h^{\left(1\right)}\\ h^{\left(2\right)} \end{array}\right]+\left[\begin{array}{c} \frac{1}{A^{\left(1\right)}}\\ 0 \end{array}\right]q^{\left(in\right)}$$

where for i=1 and 2, $h^{\left(i\right)}$ is the water level and can be calculated using the sensor’s measurements $h^{\left(i\right)}=100\times \frac{V^{\left(i\right)}}{3.3}$ where $V^{\left(i\right)}$ is voltage values measured by the input-type level transmitter placed in each tank. The flow rate $q^{\left(in\right)}$ can be calculated as $q^{\left(i\right)}=\frac{f^{\left(i\right)}}{98}$ and $q^{\left(in\right)}=\frac{f^{\left(in\right)}}{98}$ where $f^{\left(i\right)}$ and $f^{\left(in\right)}$ are measured by the flow meters. In addition, $A^{\left(1\right)}$ and $A^{\left(2\right)}$ are the cross-sectional areas of the water tanks, and $r^{\left(1\right)}$ and $r^{\left(2\right)}$ are water resistance. Furthermore, $y^{\left(1\right)}$ and $y^{\left(2\right)}$ are output variables satisfying the following relationship

(38) $$\left[\begin{array}{c} y^{\left(1\right)}\\ y^{\left(2\right)} \end{array}\right]=\left[\begin{array}{cc} 1 & 0\\ 0 & 1 \end{array}\right]\left[\begin{array}{c} h^{\left(1\right)}\\ h^{\left(2\right)} \end{array}\right].$$

Based on parameters of the experimental platform, the discretized model of the system in Equation (37) with a sample of 5 s is formulated as follows

(39) $$\left[\begin{array}{c} h_{k+1}^{\left(1\right)}\\ h_{k+1}^{\left(2\right)} \end{array}\right]=\left[\begin{array}{cc} 0.99016 & 0.0024\\ 0.0024 & 1.12047 \end{array}\right]\left[\begin{array}{c} h_{k}^{\left(1\right)}\\ h_{k}^{\left(2\right)} \end{array}\right]+\left[\begin{array}{c} 0.0145\\ 0 \end{array}\right]q^{\left(in\right)}+w_{k},$$

(40) $$\left[\begin{array}{c} y_{k}^{\left(1\right)}\\ y_{k}^{\left(2\right)} \end{array}\right]=\left[\begin{array}{cc} 1 & 0\\ 0 & 1 \end{array}\right]\left[\begin{array}{c} h_{k}^{\left(1\right)}\\ h_{k}^{\left(2\right)} \end{array}\right]+v_{k}.$$

where the noise processes $\left\{w_{k}\right\}$ and $\left\{v_{k}\right\}$ are assumed mutually independent, white, zero-mean and have known variance $Q_{w}\geq 0$ and $R_{v}>0$, respectively. The error accuracy $e_{m}$ of the level transmitters is ±0.5 centimeters. Considering the main technical specifications of water level sensors, the following parameters are chosen as $\bar{M}=1$, $Q_{w}=\left[\begin{array}{cc} 1 & 0\\ 0 & 1 \end{array}\right]\mathrm{and}\;R_{v}=\left[\begin{array}{cc} 0.25 & 0\\ 0 & 0.25 \end{array}\right]$. The nonlinear function $\bar{\phi }\left({\bar{x}}_{k}\right)=\left[\begin{array}{c} cosh_{k}^{\left(1\right)}\\ sinh_{k}^{\left(2\right)} \end{array}\right]$ and formulate the first-order expansion term coefficient $G_{k}=\left[\begin{array}{cc} -\sin{\hat{h}}_{k}^{\left(1\right)} & 0\\ 0 & \cos{\hat{h}}_{k}^{\left(2\right)} \end{array}\right]$ with the high-order expansion term $H_{k}=diag\left[\begin{array}{cc} 0.1 & 0.2 \end{array}\right]$.

### 4.3. Assessment of Effectiveness of the Theoretical Results

In this part, the effectiveness of the proposed estimator and data-forwarding scheme will be assessed through the following experiments.

**Figure 4:**
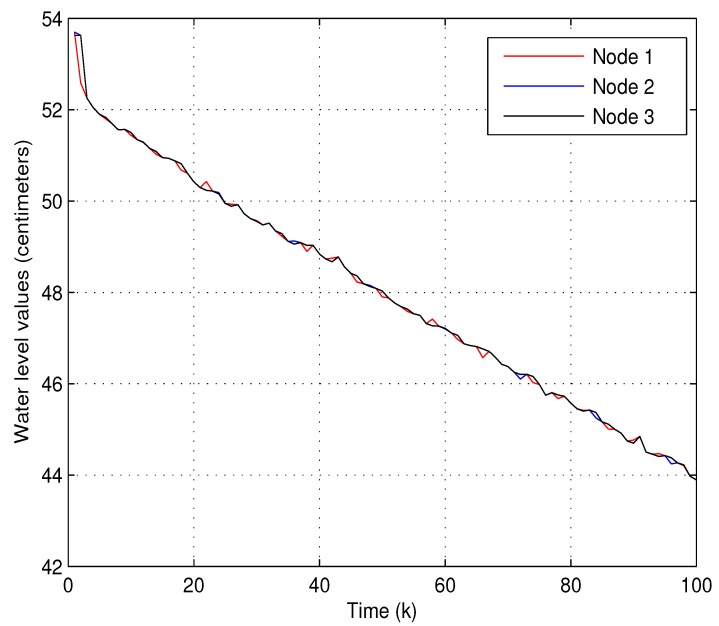
The measured and the estimated water level values for the first water tank.

**Figure 5:**
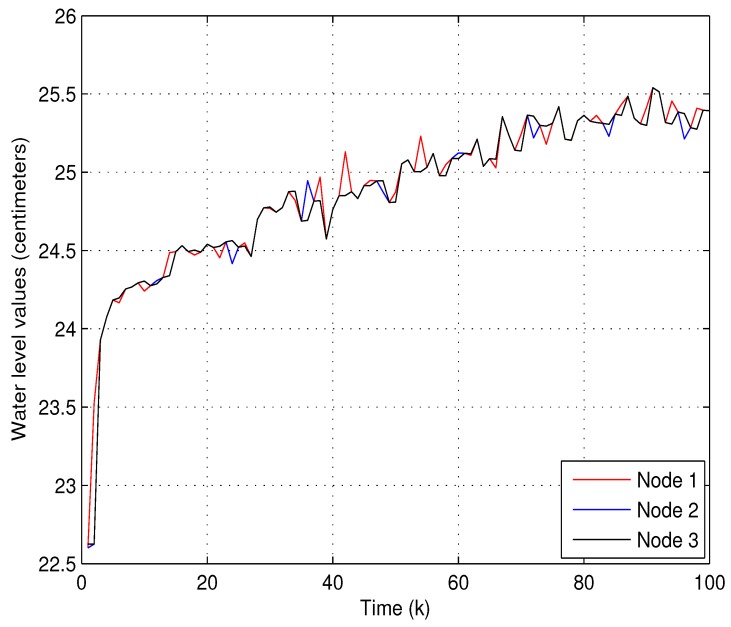
The measured and the estimated water level values for the second water tank.

**Figure 6:**
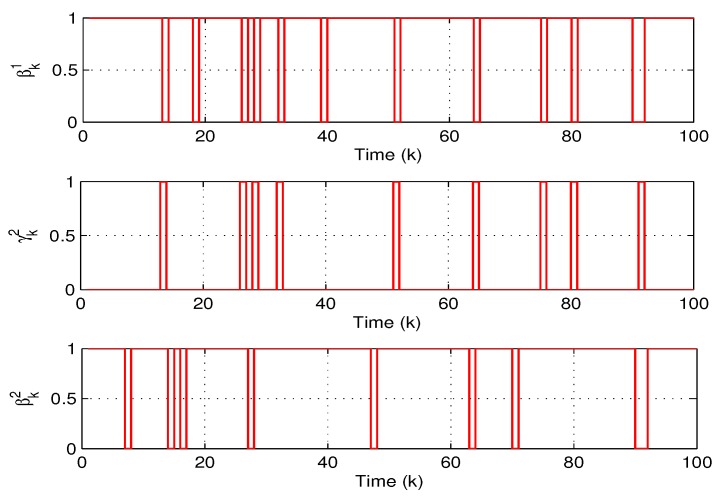
The communication behaviors on $\beta_{k}^{1}$, $\gamma_{k}^{2}$ and $\beta_{k}^{2}$.

(1) Experiment 1: In the first experiment, the accuracy of state estimation will be evaluated by using the proposed data-forwarding scheme. we temporarily ignore system fault ${\bar{f}}_{k}$ in system Equation (1) for the convenience of discussion. The running times of this system is set to 50, and the initial water level of the twin water tanks are 53 and 24 centimeters, respectively. To verify the practicability of the proposed algorithm, the following parameters are set as $\theta^{i}=\mathrm{Pr}\left\{\beta_{k}^{i}=1\right\}=0.9$ (*i* = 1 and 2), $\mathrm{Pr}\{\alpha_{k}=1\}=0.95$, γ=0.002, $L_{k}=diag\left[\begin{array}{cc} 0.01 & 0.01 \end{array}\right]$ and the transmission threshold $\delta^{i}=0.032$ (*i*= 1 and 2). Figure 4 and Figure 5 show that two water levels measured by the level transmitters and the estimated water levels of each node via our proposed estimator and data-forwarding scheme. As shown in Figure 4 and Figure 5, the measured values and the estimated values are coincident as time increases. Obviously, the estimation accuracy is satisfactory using the proposed data transmission scheme. Moreover, the corresponding communication behaviors on $\beta_{k}^{1}$, $\gamma_{k}^{2}$ and $\beta_{k}^{2}$ at each time instant are demonstrated in Figure 6. It can be also noted that our data-forwarding scheme can effectively reduce the update frequency as compared with the traditional time-triggered mechanism.

(2) Experiment 2: To verify the performance of event-triggered fault estimation, the following fault scenarios are used to complete our second experiment. A constant fault

(41) $${\bar{f}}_{k}=\left\{\begin{array}{cc} 5 & 30<k<60\\ 1 & otherwise \end{array}\right.$$

and a time-varying fault

(42) $${\bar{f}}_{k}=\left\{\begin{array}{cc} 10\sin(0.5i) & 30<k<60\\ -0.7\sin(0.5i)-0.5 & otherwise \end{array}\right.$$

**Figure 7:**
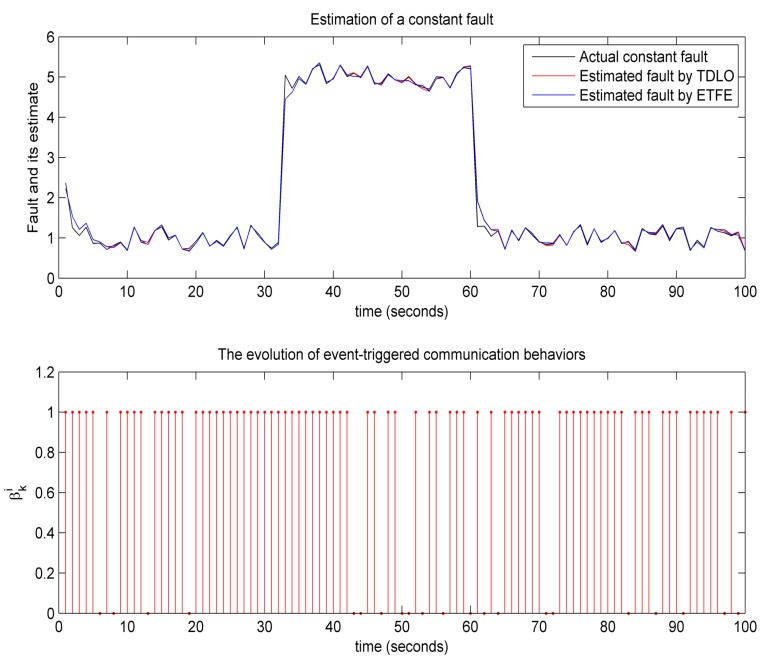
Reconstruction of a constant fault.

**Figure 8:**
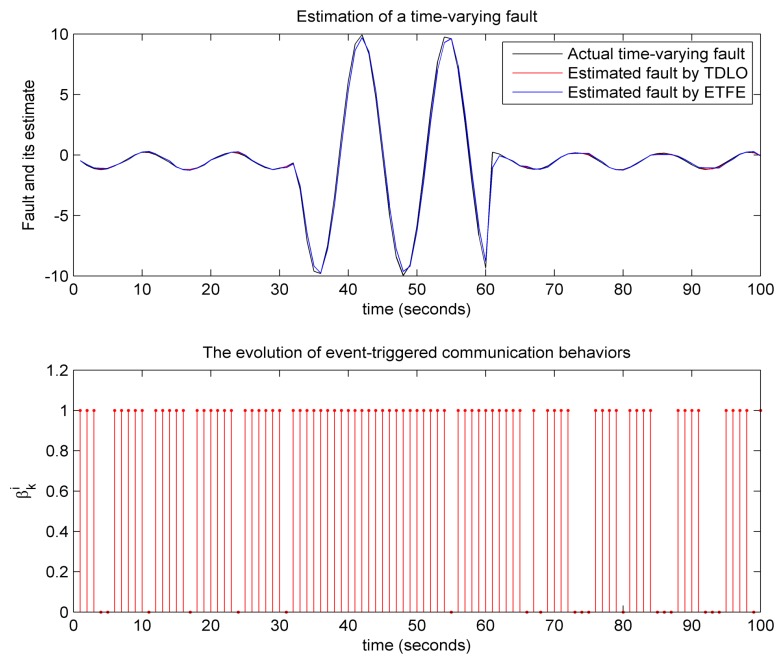
Reconstruction of a time-varying fault.

The estimated signals of a constant fault and a time-varying fault are illustrated in Figure 7 and Figure 8, respectively. As comparison, fault-estimating signals using the time-driven learning observer (TDLO) borrowed from [4] and the evolution of event-triggered communication behaviors are also depicted Figure 7 and Figure 8. It is worth mentioning that, compared with TDLO, the proposed event-triggered fault estimation (ETFE) not only provides better rapidity of fault estimation but also achieves robust reconstruction of the constant and time-varying actuator faults. Further, we examine the effect on the estimation performance from the different α and $\beta^{i}$ (*i*= 1 and 2) in Table 1 and Table 2, respectively. We can also find that a larger probability corresponds to a smaller error bound, that is, when randomly sensor nonlinearity and packet dropout have smaller probabilities of occurring, the fault estimation can achieve a better performance. All of these make it possible for the ETFE to be easily implemented in practice.

**Figure 9:**
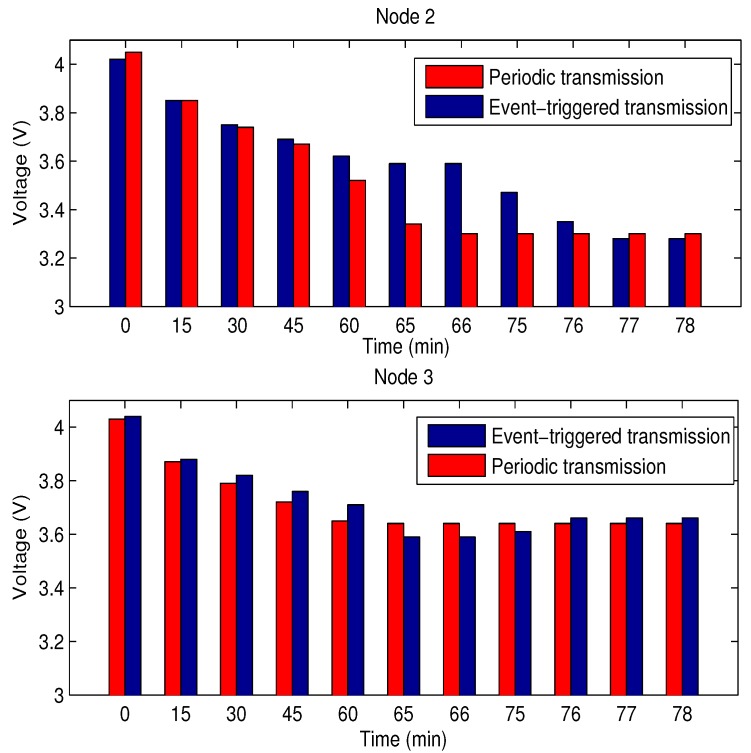
The comparison of battery voltage for Node 2 and Node 3.

(3) Experiment 3: Here, the energy conservation is now verified using a 50 mAh battery. The comparison of battery voltage at Node 2 and Node 3 are illustrated by Figure 9 where the battery voltage using the periodically forwarding scheme drops to 3.28 V after 66 min. Node 2 cannot work normally because its working voltage must exceed 3.3 V [53]. Comparatively, the battery voltage at Node 2 using the VDFS reaches 3.3 V after 77 min. We find that Node 2 consumed more energy than other nodes. The reason is that the 315M transmitter and receiver are installed at Node 2 and they can consume more energy. Because the network topology described in Figure 1 is fixed, the system stop operating once the battery at Node 2 is completely consumed. The working life of the battery is prolonged by 16.7%.

**Remark** **6.** The battery voltage for Node 1 is ignored. Because $\gamma_{k}^{0}$ can never be equal to zero constrained by the VDFS, we can utilize the sensor data transmission schedule (e.g., [31,32]) for Node 1 to achieve energy-saving in the practical applications.

## 5. Conclusions and Further Work

In this work, we have addressed the co-design problem of state and fault estimation with an event-triggered data-forwarding scheme against both randomly occurring nonlinearity and randomly occurring packet dropouts governed by Bernoulli-distributed sequences in multi-hop relay wireless networks. Recursive Riccati-like matrix equations are established to calculate the estimator gain in order to minimize an upper bound of error covariance. A Sufficient condition and a data-forwarding scheme have been derived to achieve the mean-square boundedness of the error covariance in the multi-hop relay links with random packet dropouts. Such data-forwarding scheme enables each relay node to forward the estimated values to the remote estimator. Furthermore, a new transmission protocol can be applied to the desired event-triggered transmission scheme under the fixed network topology where a relay node has the knowledge of its previous relay node and of next relay node. The effectiveness of the proposed technique has been evaluated by using a twin water-tank system with a sensor and two relay nodes.

However, we also find some open problems that should be solved in future research. First, time delays should be considered in this kind of network topology. The constant (or random) time delays can occur if the number of relays are large. Next, a switching module S9013 has been used for turning the wireless transmission module on and off. However, the wireless transmission module may reduce the operating life due to frequent opening and closing. It is necessary that the wireless transmission module implement self-dormancy for energy saving. Finally, combing event-triggered transmission scheme and coding technologies may be an interesting direction for improving energy conservation in multi-hop relays networks.

## Figures and Tables

**Table 1:** Upper bound of fault estimation error covariance with different α for $\beta^{i}=0.9$.

1−α	0.12	0.16	0.22	0.26	0.32	0.36	0.42	0.46
**An upper bound of error covariance**	0.231	0.24	0.373	0.381	0.396	0.412	0.438	0.466

**Table 2:** Upper bound of fault estimation error covariance with different $\beta^{i}$ for α=0.95.

$1-\beta^{i}$ **(*i* = 1 and 2)**	0.12	0.16	0.22	0.26	0.32	0.36	0.42	0.46
**An upper bound of error covariance**	0.315	0.362	0.397	0.416	0.478	0.503	0.612	0.681
